# Thermal tolerance and vulnerability to warming differ between populations of wild *Oncorhynchus mykiss* near the species’ southern range limit

**DOI:** 10.1038/s41598-023-41173-7

**Published:** 2023-09-04

**Authors:** T. L. Dressler, V. Han Lee, K. Klose, E. J. Eliason

**Affiliations:** 1 Department of Ecology, Evolution, and Marine Biology, University of California, Santa Barbara, CA 93106 USA; 2 U.S. Forest Service, Los Padres National Forest, 1980 Old Mission Drive, Solvang, CA 93463 USA

**Keywords:** Ecophysiology, Conservation biology, Biogeography

## Abstract

Fish habitat temperatures are increasing due to human impacts including climate change. For broadly distributed species, thermal tolerance can vary at the population level, making it challenging to predict which populations are most vulnerable to warming. Populations inhabiting warm range boundaries may be more resilient to these changes due to adaptation or acclimatization to warmer temperatures, or they may be more vulnerable as temperatures may already approach their physiological limits. We tested functional and critical thermal tolerance of two populations of wild *Oncorhynchus mykiss* near the species’ southern range limit and, as predicted, found population-specific responses to temperature. Specifically, the population inhabiting the warmer stream, Piru Creek, had higher critical thermal maxima and higher functional thermal tolerance compared to the population from the cooler stream, Arroyo Seco. Arroyo Seco *O. mykiss* are more likely to experience a limitation of aerobic scope with warming. Piru Creek *O. mykiss*, however, had higher resting metabolic rates and prolonged exercise recovery, meaning that they could be more vulnerable to warming if prey or dissolved oxygen become limited. Temperature varies widely between streams near the *O. mykiss* southern range limit and populations will likely have unique responses to warming based on their thermal tolerances and metabolic requirements.

## Introduction

As global temperatures continue to rise due to climate change, aquatic ectotherms are projected to undergo increased local extinctions at warm range boundaries, critically altering the distribution and population dynamics of vital fish stocks^1–3^. Poleward range shifts have already been observed in both marine^4^ and freshwater^5^ fish species, as populations inhabiting extreme conditions at warm range boundaries are likely to experience physiologically limiting temperatures^6–8^. However, exposure to such conditions over generations can lead to local adaptation, allowing these fringe populations to be better equipped to withstand temperature extremes^9–12^. To predict species range shifts and to effectively manage populations at range edges, it is important to understand their vulnerability to current conditions, and their potential resiliency to ongoing climate-induced temperature change.

Vulnerability and resiliency to current and future habitat temperatures can be assessed by measuring functional and critical thermal limits. Functional thermal tolerance limits refer to temperatures where key fitness-related performance traits become restricted. At these temperatures, fish do not die but are limited in their ability to grow, compete, evade predators, and/or reproduce^13–16^. Functional thermal tolerance is often approximated by measuring aerobic capacity across a range of temperatures because many of these essential performance traits are dictated by aerobic metabolism^16,17^. Optimal and limiting temperatures are often mediated by the amount of oxygen fish have available after accounting for baseline requirements, which tend to increase exponentially with rising temperature^18,19^. Aerobic capacity, or aerobic scope, can be quantified by calculating the difference (absolute aerobic scope, AAS) or the ratio (factorial aerobic scope, FAS) between oxygen consumption rates of individual fish at rest (resting metabolic rate, RMR) and during or immediately after maximal exercise (maximum metabolic rate, MMR). This concept of oxygen and capacity limited thermal tolerance (OCLTT)^20–22^ is disputed and the relationships between temperature, aerobic scope, and other whole animal performances can vary between species and temperature regimes^23–26^. Nevertheless, optimum and limiting temperatures for aerobic scope have been linked to fish range limits^27^. In addition, the ability to recover from exhaustive exercise is crucial for wild fish as they frequently use anaerobic burst swimming (e.g. foraging, avoiding predators, interacting with fisheries, competing with conspecifics for space or mates). Prolonged recovery could lead to lost opportunities (e.g. food, space, mates) or increased vulnerability (e.g. predators, disease). The effect of temperature on the speed of recovery from MMR and on the amount of oxygen required to recover from MMR (excess post-exercise oxygen consumption; EPOC) can therefore also be used as a metric of functional thermal tolerance^19^.

Critical thermal limits are temperatures that are lethal to fish when temperature is increased or decreased rapidly. Critical thermal maximum (CT_MAX_) tests approximate upper lethal temperatures by measuring loss of equilibrium^28^. Fish are unlikely to survive loss of equilibrium in the wild, especially in the presence of predators^29^. There is an apparent latitudinal gradient in fish CT_MAX_ where species occupying warmer habitats at lower latitudes tend to have the highest CT_MAX_^30^, but CT_MAX_ can also vary within species at the population level and can change at the individual level depending on holding or habitat temperature (i.e., acclimation/acclimatization), time of day, and heating rate^28,31–33^. CT_MAX_ are used to calculate thermal safety margins (TSMs), or the difference between the maximum habitat temperature and the upper lethal temperature^34,35^. Because temperature tends to limit fish performance at temperatures below CT_MAX_, however, TSMs often overestimate the amount of warming that a fish population can withstand before facing declines^14,36,37^. Therefore, functional thermal tolerance limits are used to calculate functional warming tolerance (FWT), or the difference between maximum habitat temperature and the temperature where fish performance begins to decline, and this can be used to understand the relative vulnerabilities of fish populations to habitat warming^37,38^.

Fishes that inhabit broad geographic ranges often consist of genetically distinct populations that experience vastly different thermal conditions. Acclimatization or local adaptation to a range of thermal habitats leads to interpopulation variability in thermal tolerance^11,12,39–41^. In warm environments, specific adaptive or acclimation strategies include enhanced heat shock protein production^31,40,42^, increased surface area of gill lamellae^43,44^, enhanced cardiac capacity (due to increased heart size, compact myocardium thickness and capillary density)^39,45–48^, and increased mitochondrial capacity^49,50^. These responses, whether plastic or adaptive, can boost a population’s ability to tolerate temperature extremes^11,12^. They also enable physiological processes, particularly those dictated by aerobic metabolism, to be optimized at habitat-specific temperatures^39,46,51^. However, maintaining adaptations and mounting acclimatory responses are energetically costly and can have fitness consequences for warm-exposed fish populations^52^. These costs also frequently result in a tradeoff between upper thermal tolerance and thermal plasticity where populations with the ability to tolerate high temperatures have restricted capacity for thermal acclimation and vice versa^53,54^.

Wild Pacific salmon, of the genus *Oncorhynchus*, are of critical ecological, cultural, and economic importance and many species have already experienced declines due to increased water temperature combined with habitat degradation and increased frequency and intensity of drought^55–57^. Declines are especially prominent near the warm range boundaries for these species^58^. *Oncorhynchus mykiss* (steelhead/rainbow trout), *Oncorhynchus kisutch* (coho salmon), and *Oncorhynchus tshawytscha* (chinook salmon), for example, are considered endangered or threatened in coastal California under the United States Federal Endangered Species Act. Pacific salmonid populations currently persisting at these range limits may already experience physiologically limiting temperatures and are likely vulnerable to further declines and extirpation should temperatures continue to increase. We studied wild *O. mykiss*, also known as steelhead (anadromous phenotype) or rainbow trout (freshwater resident phenotype) that inhabit an extraordinarily broad native distribution, extending along the west coast of North America from Baja California to Alaska, and across to the Kamchatka Peninsula in eastern Russia^59^. Although these fish are most often reported to occupy cold-water habitats, *O. mykiss* populations inhabit a wide range of thermal conditions across their latitudinal distribution. Thermal tolerance has been studied in this species in the central part of its range (e.g., Northern California^60^, Central California^51^), but thermal tolerance has never been studied in wild *O. mykiss* inhabiting their southern range limit. We predicted that *O. mykiss* inhabiting their southern range limit have distinctive adaptations to cope with warm temperatures due to selection pressures associated with their habitat conditions.

**Figure 1:**
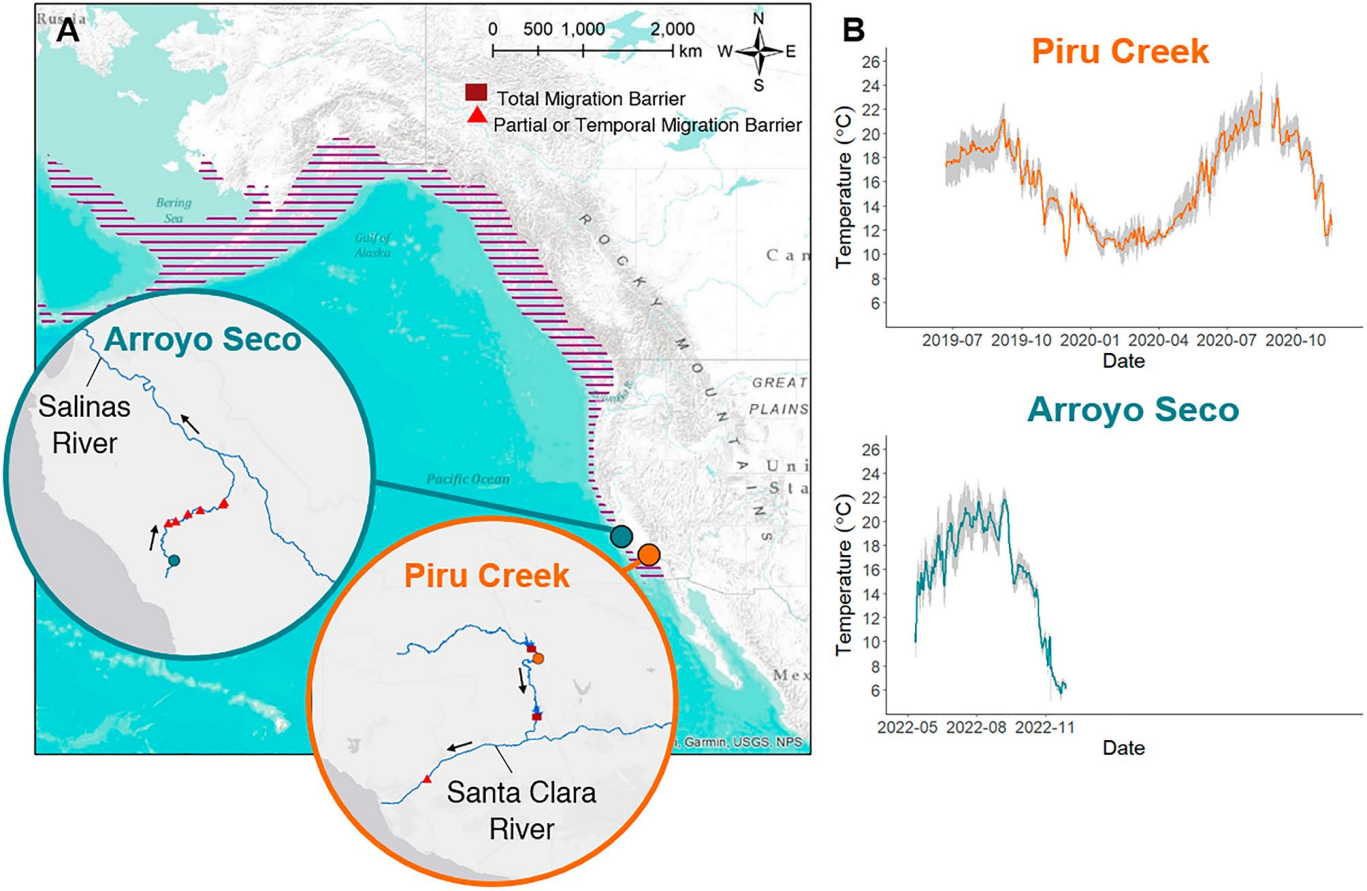
Location of study populations (**A**), Arroyo Seco (blue) and Piru Creek (orange), and temperatures at each site (**B**). Circles indicate each study site location. Purple hatched shading shows the full range of anadromous *O. mykiss* in North America^98^. Dark red rectangles indicate impassable barriers to fish migration (dams) and red triangles indicate partial or temporal barriers to migration (e.g., road crossings and other human-constructed or natural features that require high flow for fish passage). Black arrows indicate flow direction. Stream temperatures were recorded at 15-min intervals using Onset Pendant loggers. Orange and blue lines indicate mean daily stream temperatures and gray shading represents minimum and maximum daily temperatures.

In this study, we aimed to determine whether wild *O. mykiss* near their warm range boundary exhibit interpopulation variability in thermal tolerance and whether these populations currently experience physiologically limiting temperatures or if they could withstand additional warming. We measured aerobic capacity, exercise recovery, and CT_MAX_ in two genetically distinct^61^ populations of wild *O. mykiss* that inhabit distinctly different thermal habitats near the southern range limit for the species (Fig. 1). We conducted all experiments streamside using ecologically relevant diurnally cycling temperatures to mimic natural environmental conditions^38^. In addition, we collated stream temperature data from 11 trout-bearing streams in the Los Padres National Forest in Southern California to assess temperature regimes experienced by trout at their range limit. We predicted that the population from the warmer stream, Piru Creek, would have a higher upper thermal tolerance and a reduced capacity to rapidly acclimate to elevated temperatures than the population from the cooler stream, Arroyo Seco. We also predicted that the Piru Creek population would have reduced TSM and FWT due to higher habitat temperatures and would thus be less capable of withstanding future warming than the Arroyo Seco population.

## Results

### Critical thermal maximum

**Figure 2:**
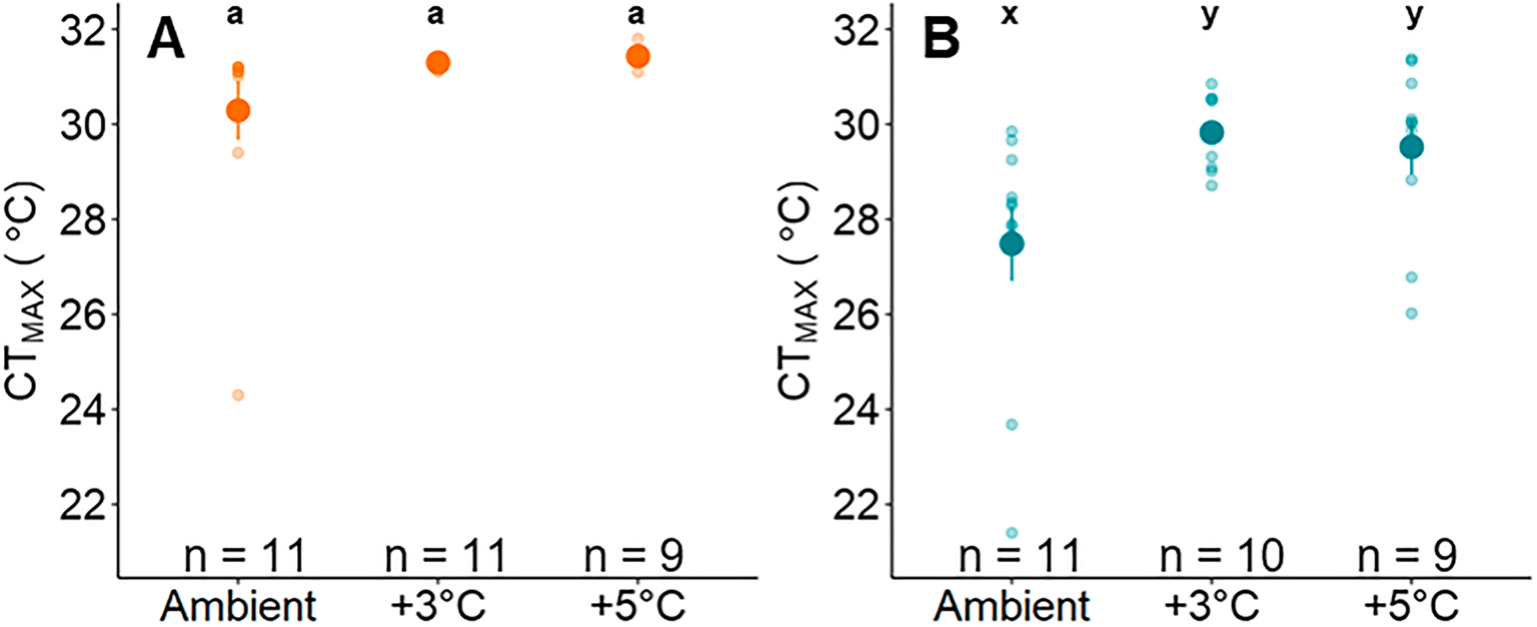
Critical thermal maximum (CT_MAX_) for each temperature treatment at Piru Creek (**A**) and Arroyo Seco (**B**). Bold lines indicate median CT_MAX_ for each treatment. Differing letters indicate statistically significant differences within populations (one-way Anova; p < 0.05; Piru Creek: a, b; Arroyo Seco: x, y).

CT_MAX_ was higher, but less plastic for *O. mykiss* from Piru Creek compared to the Arroyo Seco. At Arroyo Seco, mean CT_MAX_ ranged from 27.5 to 29.8 °C and increased significantly with holding temperature (Fig. 2B; Table S1). At Piru Creek, CT_MAX_ averaged 31.0 °C and did not differ among temperature treatments (Fig. 2A; Table S1). When comparing common temperature treatments between populations, Piru Creek *O. mykiss* had a higher CT_MAX_ than Arroyo Seco *O. mykiss* (Table S2).

### Oxygen uptake rate

**Figure 3:**
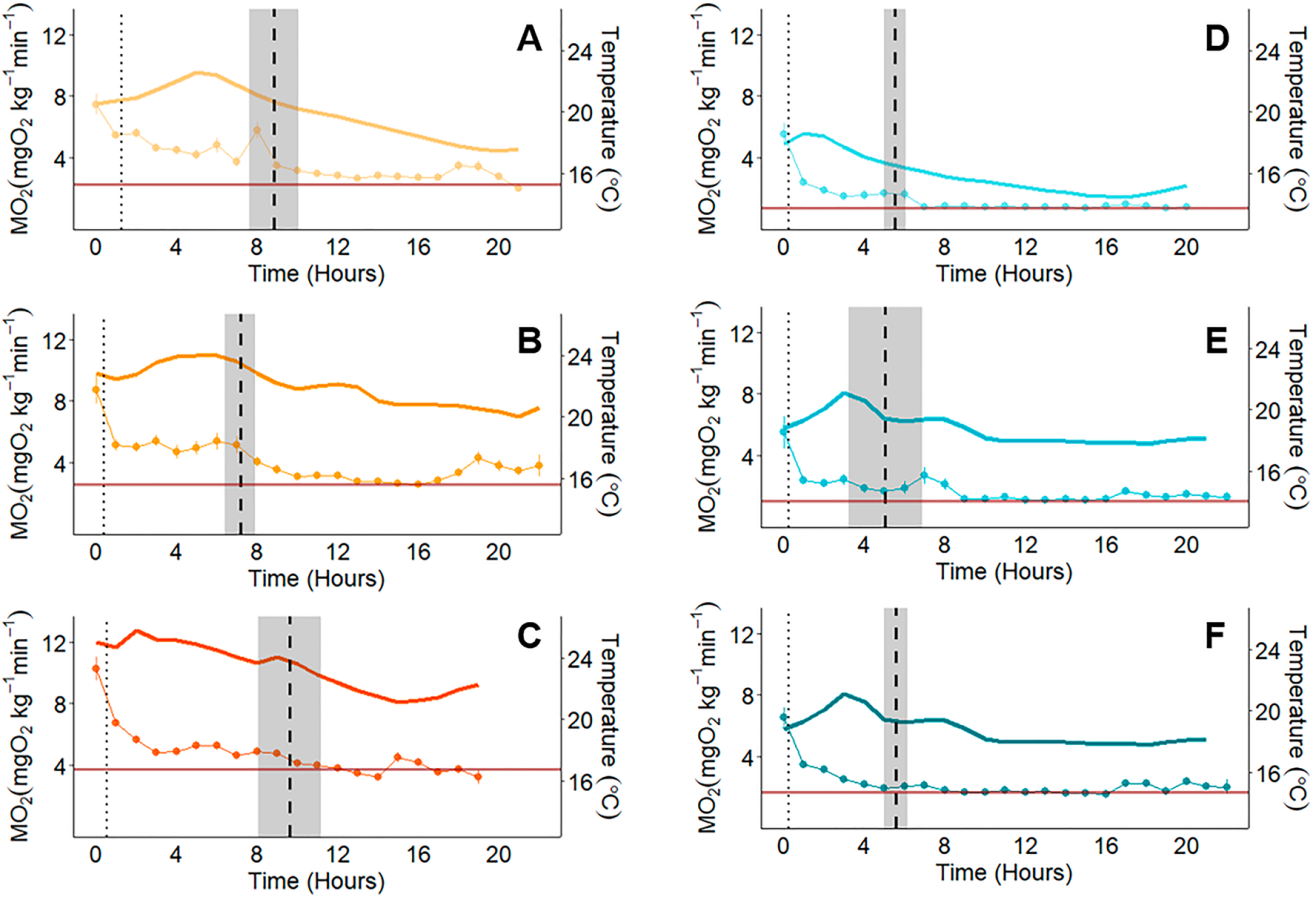
Mean ± SEM hourly oxygen uptake rate (MO_2_) and temperature over the duration of the experiment for each temperature treatment (**A** and **D**: Ambient, **B** and **E**: + 3 °C, **C** and **F**: + 5 °C) and population (Piru: orange, Arroyo Seco: blue). The temperature profile that occurred during the experiment is indicated by the solid-colored lines. Each point represents the mean ± SEM hourly MO_2_ for the fish in each respective group. The data point at time 0 was taken immediately after the chase and represents Maximum Metabolic Rate (MMR). Mean standard metabolic rate (SMR; red horizontal lines), mean time to reach 50% of MMR (MMR50; vertical dotted lines) and the mean ± SEM time to reach 20% of SMR (duration of recovery, i.e., duration of EPOC; vertical dashed lines) are indicated.

Following the chase, all fish exhibited a similar oxygen uptake rate (MO_2_) profile characterized by a rapid drop in MO_2_ after maximum metabolic rate (MMR) followed by a slow decline in MO_2_ to a stable, baseline level. Within treatments, resting metabolic rate (RMR) remained stable despite fluctuating temperatures (Fig. 3).

For both populations, MMR increased with increasing temperature (Table 1). MMR was not measured at common temperatures between populations so we compared MMR measurements between similar temperatures: Arroyo Seco at 19 °C and 21 °C and Piru Creek at 20 °C. There were no significant differences in MMR between populations at these temperatures (df = 2, F = 0.068, p = 0.935).

**Table 1:** Oxygen uptake rate data [Maximum metabolic rate (MMR); Standard Metabolic Rate (SMR); Resting Metabolic Rate (RMR) at aerobic scope temperatures; Absolute Aerobic Scope (AAS), Factorial Aerobic Scope (FAS)] for each temperature treatment and population, all values are presented as mean ± SEM.

Population	Group	Holding temperature range (°C)	MMR temperature (°C)	n (MMR)	MMR	n (SMR, RMR, AAS, FAS)	SMR	RMR temperature (°C)	RMR	AAS	FAS
Piru Creek	Ambient	17–22	20	8	10.19 ± 1.06^a^	6	2.37 ± 0.16^a^	19	2.69 ± 0.15	7.60 ± 1.16	3.78 ± 0.34
	+ 3 °C	20–24	23	9	12.71 ± 1.04^ab^	6	2.42 ± 0.29^a^	22	3.38 ± 0.46	9.64 ± 1.34	4.05 ± 0.453
	+ 5 °C	21–26	25	8	14.34 ± 0.72^b^	5	3.23 ± 0.16^b^	23	3.70 ± 0.26	10.70 ± 0.51	3.97 ± 0.32
Arroyo Seco	Ambient	14–19	18	10	7.43 ± 0.67^x^	5	0.76 ± 0.11^x^	16	0.83 ± 0.15	7.35 ± 0.84	10.48 ± 1.24^x^
	+ 3 °C	17–21	19	7	10.25 ± 1.15^y^	5	1.62 ± 0.38^xy^	19	1.72 ± 0.43	9.03 ± 1.15	6.76 ± 0.52^y^
	+ 5 °C	19–24	21	8	10.64 ± 0.59^y^	6	1.94 ± 0.30^y^	21	2.03 ± 0.19	9.29 ± 0.29	5.77 ± 0.44^y^

Differing letters indicate statistically significant differences within populations (one-way Anova; p < 0.05; Piru Creek: a, b; Arroyo Seco: x, y).

**Figure 4:**
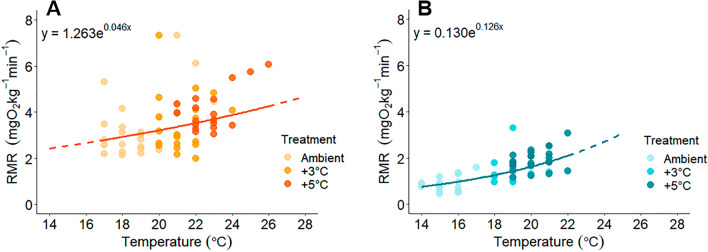
Resting metabolic rate (RMR) measurements for each temperature at Piru Creek (**A**) and Arroyo Seco (**B**). Solid lines indicate predicted values within the range of measured RMR temperatures and dashed lines indicate predicted values outside of the range of measured RMR temperatures.

**Figure 5:**
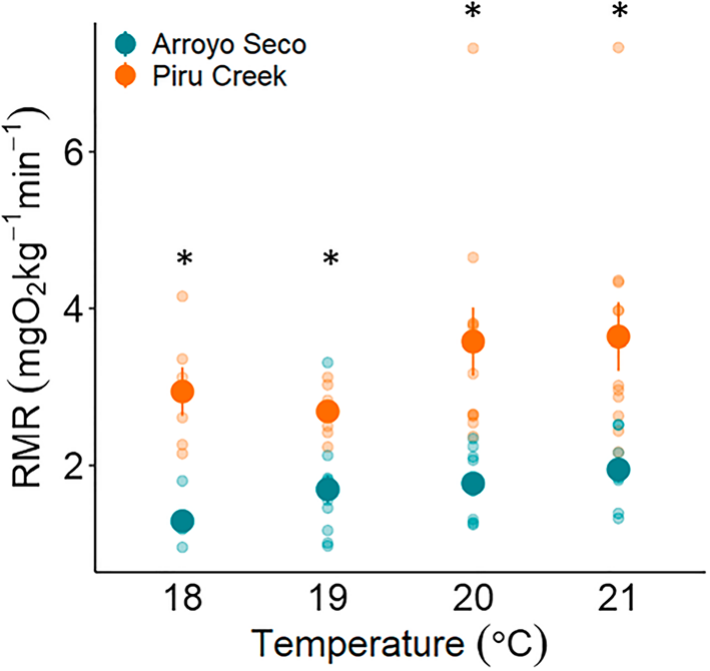
Resting metabolic rate (RMR) for each population at 4 common temperatures. Asterisks indicate statistically significant differences between populations at each temperature (t-test or Welch’s t-test; p < 0.05).

RMR increased exponentially with increasing temperature at both populations (Fig. 4; model selection Table S3). RMR was measured at four common temperatures between the two populations (18 °C, 19 °C, 20 °C, and 21 °C; Fig. 5, Table S4). At Piru Creek, RMR of *O. mykiss* was 1.6–2.3 times greater than RMR in trout from Arroyo Seco at all common temperatures (Table S4; Fig. 5). Notably, between 18 and 21 °C, RMR of Arroyo Seco *O. mykiss* had a greater Q_10_ (3.95) compared to Piru Creek (2.03) signifying that RMR of Arroyo Seco trout is more temperature sensitive than RMR of Piru Creek trout.

**Figure 6:**
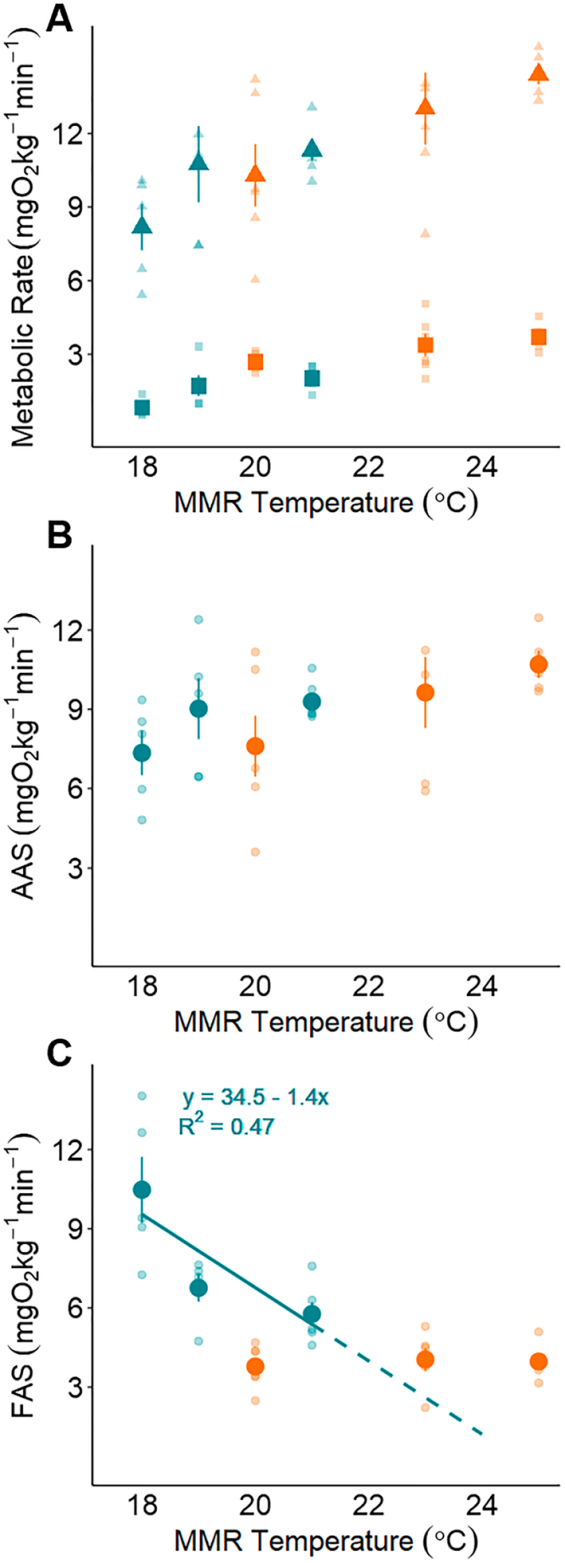
Maximum metabolic rate (MMR; triangle symbols; **A**), resting metabolic rate (RMR; square symbols; **A**), absolute aerobic scope (AAS; **B**) and factorial aerobic scope (FAS; **C**) for individual fish (small points) and mean ± SEM for each temperature treatment (large points) for Piru Creek (orange symbols) and Arroyo Seco (blue symbols). Modeled relationship between temperature and FAS for Arroyo Seco is represented by the line and the corresponding equation. See Table 1 for sample size.

Absolute aerobic scope (AAS) was not significantly different across test temperatures for either population (Piru Creek: df = 2, F = 1.652, p = 0.227; Arroyo Seco: df = 2, F = 1.742, p = 0.214; Table 1). The relationship between temperature and factorial aerobic scope (FAS), however, was population dependent. At Piru Creek, FAS did not vary significantly with temperature. At Arroyo Seco, FAS declined significantly with increasing temperature (Table 1, Fig. 6). At all test temperatures, FAS remained above 3 at both populations. Temperatures where FAS ≤ 3 are thought to be associated with feeding and growth limitations^19,37,62,63^, because RMR tends to increase by 2–3 times during digestion. We therefore aimed to identify the temperatures where FAS = 3 for each population. Based on a linear model fit from these data, we predict that FAS would reach 3 for the Arroyo Seco population at ~ 23 °C (Fig. 6). This metric could not be estimated for the Piru Creek population because we were unable to fit a model describing the relationship between FAS and temperature.

### Recovery

**Figure 7:**
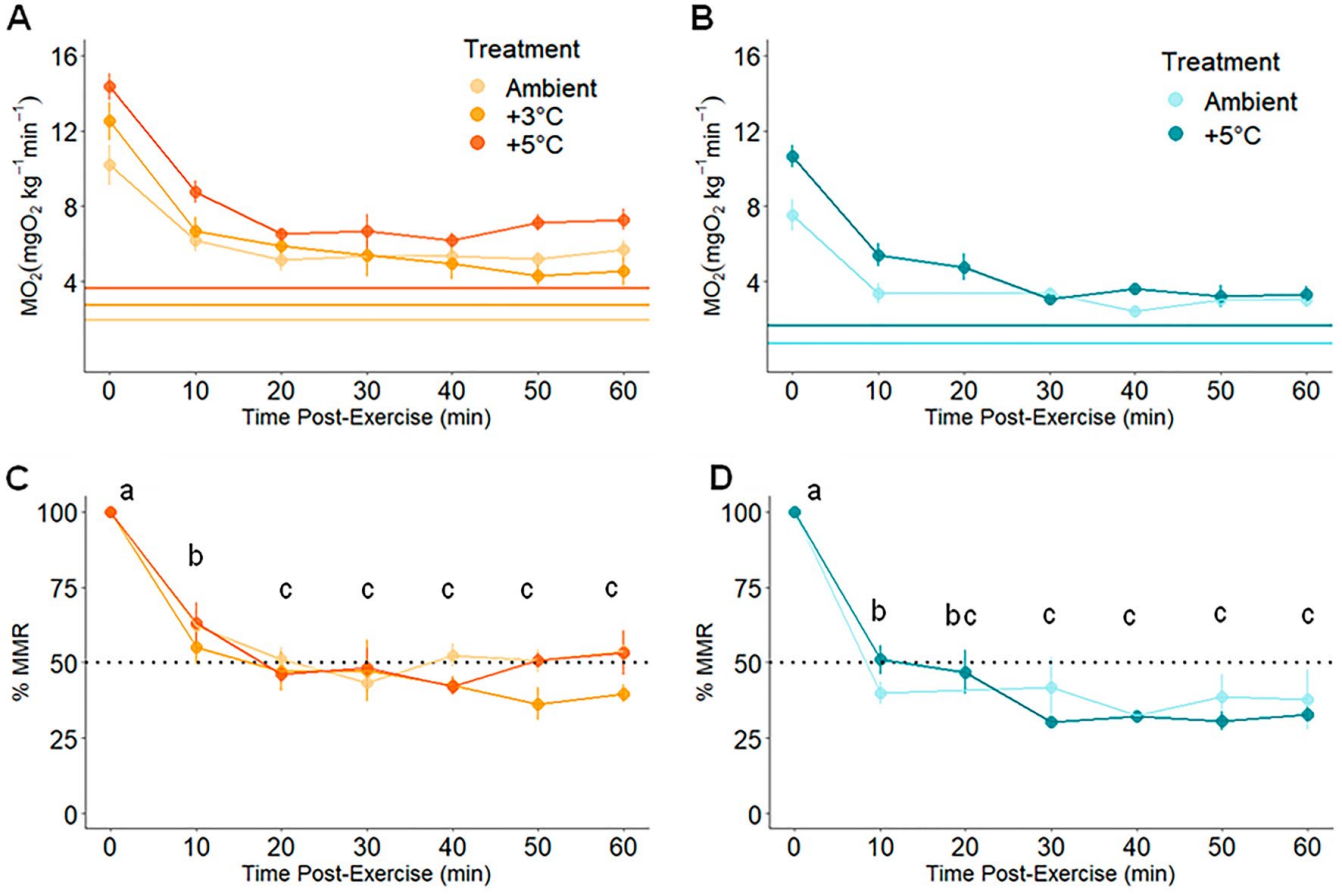
Oxygen uptake rates (MO_2_) over the first hour of recovery from exhaustive exercise for Piru Creek (**A**, **C**) and Arroyo Seco (**B**, **D**) fish. Panels (**A**) and (**B**) show MO_2_ values with horizontal lines indicating the mean standard metabolic rate (SMR) for each temperature treatment. Panels C and D show MO_2_ as a percentage of maximum metabolic rate (MMR). Data is presented as mean ± SEM, lowercase letters indicate statistical differences between time points (repeated measures Anova; p < 0.05). See Table 2 for sample size.

The magnitude and duration of excess post-exercise oxygen consumption (EPOC) were higher for the Piru Creek population than for the Arroyo Seco population (magnitude: t = − 5.47, df = 22.337, p < 0.001; duration: t = − 3.54, df = 30.58, p = 0.001). EPOC magnitude averaged 416.97 ± 35.56 mg O_2_ kg^−1^ for Arroyo Seco *O. mykiss* and 1066.07 ± 111.83 mg O_2_ kg^−1^ for Piru Creek *O. mykiss*. EPOC duration averaged 517.0 ± 44.3 min for Piru Creek and 325.53 ± 31.1 min for Arroyo Seco (Fig. 3). There was no significant difference in EPOC duration or magnitude between temperature treatment groups for either population (Table 2). During the first hour of recovery, MO_2_ decreased significantly during the first 20 min of recovery and then plateaued for the next 40 min of recovery (Fig. 7). At all post-MMR time points (10, 20, 30, 40, 50 and 60 min), MO_2_ for Arroyo Seco *O. mykiss* was at a lower %MMR than Piru Creek (i.e., Arroyo Seco trout recovered their aerobic capacity more quickly than Piru Creek trout; Table S5).

**Table 2:** Excess post exercise oxygen consumption (EPOC) duration and magnitude (mean ± SEM) from each population as well as one-way ANOVA results (p < 0.05) testing for differences between temperature treatment groups within each population.

Population	Group	n (EPOC)	EPOC duration (min)	df	F-value	p-value	EPOC magnitude (mgO_2_kg^-1^)	df	F-value	p-value
Piru Creek	Ambient	6	53.1 ± 22.0	2	0.976	0.398	958.82 ± 222.52	2	0.292	0.75
	+ 3 °C	6	20.9 ± 4.9				1048.62 ± 206.55			
	+ 5 °C	7	23.0 ± 5.8				1172.96 ± 180.00			
Arroyo Seco	Ambient	5	14.6 ± 4.4	2	0.14	0.871	358.57 ± 62.84	2	1.741	0.217
	+ 3 °C	4	24.7 ± 3.0				361.02 ± 89.79			
	+ 5 °C	6	22.0 ± 3.4				502.93 ± 51.31

### Stream temperatures, thermal safety margins and functional warming tolerance

From 2019 to 2022, we measured a maximum stream temperature of 25 °C at Piru Creek. *O. mykiss *at Piru Creek therefore had a TSM of 6 °C. We cannot calculate FWT for Piru Creek *O. mykiss* because their FAS never got below 3 for any of our treatments and we could not fit a linear regression to the data to approximate the FAS = 3 temperature. We do not have continuous temperature data for Arroyo Seco during the time that we conducted these experiments because a fire and debris flow washed away our temperature loggers. During the experiments in September of 2019, the maximum stream temperature was 18 °C. After this fire and debris flow, the stream habitat is now shallower with less riparian cover and temperatures are expected to be warmer than in 2019. In the summer of 2022, we measured a maximum stream temperature of 23 °C at Arroyo Seco. Based on the mean CT_MAX_ measured for the 19–24 °C treatment, Arroyo Seco *O. mykiss* have a TSM of 6.5 °C. Based on the modeled FAS = 3 temperature for this population, we estimate that these fish have FWT of 0 °C, meaning that they currently experience temperatures at the edge of their functional thermal limits. More temperature data will be required to determine if Arroyo Seco will consistently reach or exceed 23 °C in future years.

**Figure 8:**
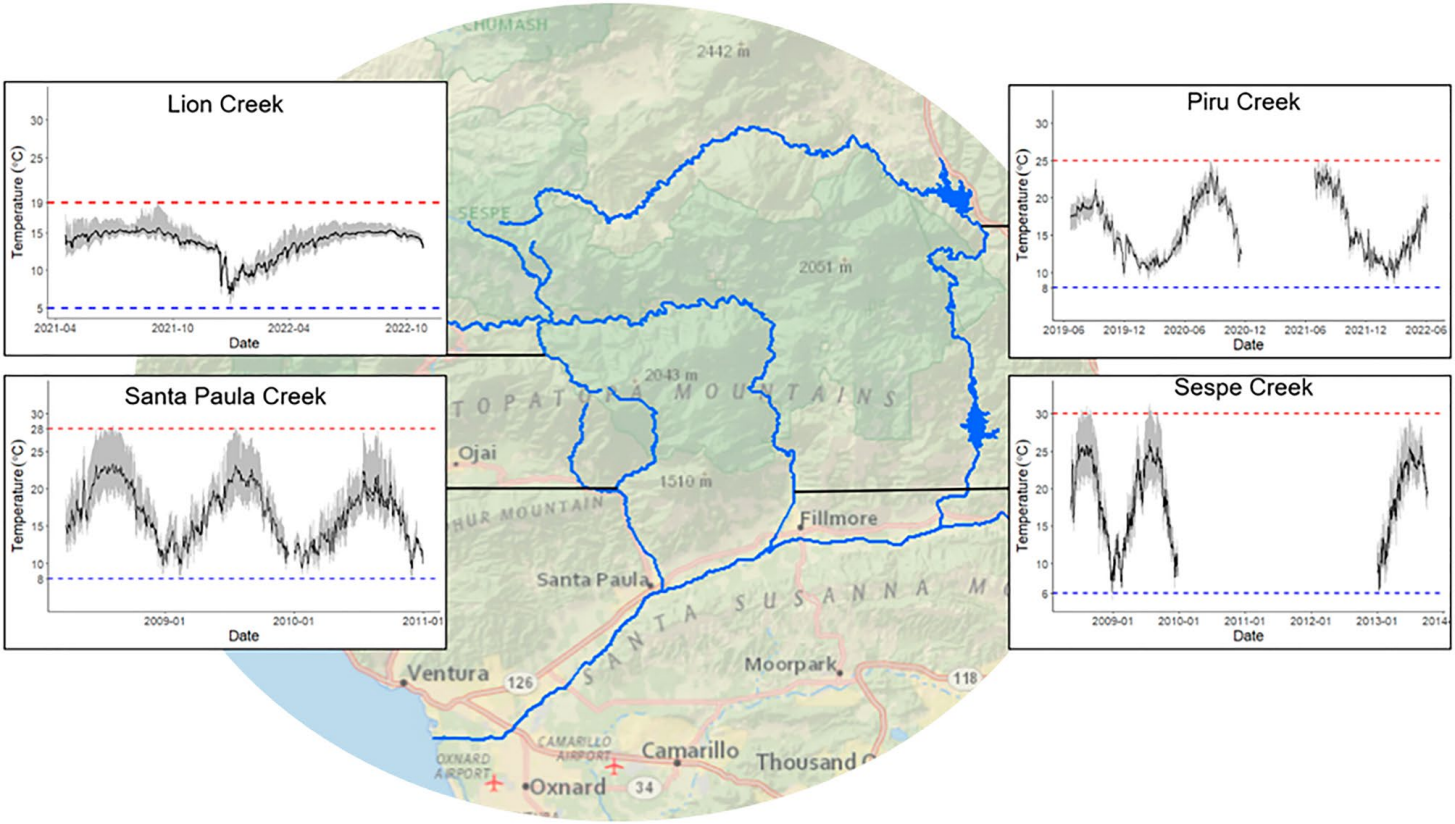
Locations of temperature data loggers in the Santa Clara River watershed with temperature data plotted for each location. In the temperature plots, black lines indicate mean daily temperatures, gray shading represents daily temperature ranges, red dashed lines indicate maximum stream temperatures, and blue dashed lines indicate minimum stream temperatures during the time data was recorded.

Water temperature regimes are highly variable within and between watersheds in the Los Padres National Forest (Figs. 8 & S3, Table S6). Based on long term temperature data from our data loggers and from open access data collected in the past, stream temperatures can get as low as 5 °C during the winter and up to 30 °C in the summer throughout the forest (Fig. 8). In the summer, some streams remain stable throughout the day with daily temperature fluctuations of 0.3–3 °C (e.g., Lion Creek, Figs. 8 & Fig. S3; Davy Brown Creek, Fig. S3; Bear Creek, Fig. S3) while others undergo daily temperature fluctuations of up to 10 °C (e.g., Santa Paula Creek, Fig. 8; Matilija Creek, Fig. S3).

## Discussion

In this study we tested the functional and critical thermal tolerance of two genetically distinct^61^ wild *O. mykiss* populations near the southern limit of their native range (Fig. 1). Southern California trout populations experience high temperatures and have undergone local declines from habitat loss and degradation^64^. We discovered that *O. mykiss* populations differ in both functional and critical thermal tolerance over a brief time scale of thermal exposure, and current local maximum temperatures exceed functional limits for the Arroyo Seco River population. Moreover, water temperatures in trout-bearing streams throughout Southern California can reach exceptionally high temperatures (e.g., 25–30 °C), and trout in this part of their range are likely confined to a limited number of thermal refugia (e.g. Lion Creek, Davey Brown Creek; Fig. S3). Southern *O. mykiss* have unique thermal physiology. We specifically chose a brief overnight temperature exposure duration representing a short-term thermal stress for these fish because it is ecologically relevant given the natural stochasticity and speed of temperature change in these systems. Full physiological acclimation to a new temperature regime can take several weeks^65^ and thus maximal acclimation capacity could not be assessed here. Even still, there is evidence that salmonids can acclimate rapidly on timescales that are similar to rates of temperature change in their wild habitats (e.g., brown trout swimming performance, 48 h^66^; Arctic char heartrate, 3 days^67^). Given the rapid temperature changes experienced in these fringing aquatic environments, fish in the wild would rarely have the time and exposure conditions (i.e. several weeks under a particular thermal condition) to fully physiologically acclimate. In agreement with our hypotheses, we found evidence of population specificity of thermal tolerance, where the population from the warmer stream (Piru Creek) has higher thermal tolerance compared to the population from the historically cooler stream (Arroyo Seco). This is unsurprising given that hatchery strains of this species have exhibited elevated thermal tolerance when exposed to warm temperatures over generations^63,68^. Piru Creek *O. mykiss* have a higher, but less plastic upper lethal temperature limit (CT_MAX_) compared to the Arroyo Seco population. Moreover, we estimated that Arroyo Seco *O. mykiss* would reach a functional thermal threshold (FAS = 3) at ~ 23 °C, while Piru Creek *O. mykiss* did not show evidence of a decline in FAS at temperatures up to 25 °C, indicating a higher functional thermal limit for the Piru Creek population. Piru Creek *O. mykiss* also appear to have a higher functional thermal tolerance compared to a warm-adapted population inhabiting the California Central Valley where FAS = 2 at 23 °C^51^ and compared to a hatchery strain that has undergone generations of selection for high thermal tolerance in Western Australia, where FAS ≤ 1.8 at 25 °C^68^. Both *O. mykiss* populations in the present study have high functional thermal tolerance compared to hatchery-raised *O. mykiss* in more northern parts of their wild range. Myrick and Cech observed diminished growth rates above 19 °C in two strains of *O. mykiss* from Northern California^60^. Many more studies have been conducted on hatchery *O. mykiss* in British Columbia, Canada and at similar latitudes in Europe. When acutely exposed to elevated temperatures, Anttila et al. found optimal aerobic and cardiac performance at 19 °C and a significant decline in performance at 23 °C in *O. mykiss*^69^. Heath and Hughes detected diminished heart function and venous oxygen deficiency in *O. mykiss* acutely exposed to 24–25 °C^70^. Following acclimation, Jain and Farrell observed impaired exercise recovery at 15 °C, Farrell et al. found diminished cardiac function at 18 °C and 22 °C, and Taylor et al. detected reduced aerobic swimming capacity at 18 °C^71–73^. There appears to be a latitudinal gradient of functional thermal tolerance for *O. mykiss* and that Piru Creek exhibit the highest functional thermal limits of any measured population.

RMR of Piru Creek and Arroyo Seco *O. mykiss* did not change significantly based on time of day or diurnal temperature (Fig. 3). In other words, the baseline oxygen requirements of these fish remained stable throughout daily temperature fluctuations of 4–5 °C. This contrasts with other salmonid species such as Atlantic salmon^74^ and coastal cutthroat trout^38^ that show clear fluctuations in RMR during ecologically relevant temperature fluctuations. These fish likely possess adaptations that allow them to withstand daily temperature fluctuations without major changes in oxygen requirements. This could be advantageous because it allows for unrestricted aerobic scope (and therefore a better ability to flee predators, hunt for food, increase growth rates and fecundity, etc.) during the warmest times of day. The consistently higher Piru Creek RMR (almost double that of Arroyo Seco fish) was an unexpected result given that warm-adapted fish populations tend to display lower RMR due to thermal compensation^31,44,75^. This suggests that the high thermal tolerance of the Piru Creek population is conferred by strategies that are energetically costly to maintain. Since peak temperatures are stochastic and relatively short-lived in this system (Fig. 1), it is possible that temperature-based selection is acting on pathways involved in acute thermal tolerance (e.g., heat shock protein production). Future studies should investigate the mechanisms for this apparent tradeoff in thermal tolerance and resting oxygen requirements.

Anaerobic activity (i.e., maximal exercise) is common in juvenile salmonids, as they continuously avoid predators and compete for space and food. Fast recovery allows them to resume their normal functions quickly after these encounters and the fast recovery times observed in this study (~ 10–20 min) are likely advantageous for this life stage. Although exercise recovery was unimpacted by test temperatures in the present study, all of our recovery metrics indicate that Piru Creek *O. mykiss* have greater costs associated with exercise recovery compared to Arroyo Seco *O. mykiss*. After MMR, the Arroyo Seco population recovered a greater proportion of their aerobic capacity in a shorter period of time than the Piru Creek population (Fig. 7). The Arroyo Seco population also had a lower EPOC magnitude and duration compared to the Piru Creek population.

CT_MAX_ experiments were conducted after fish had been exposed to their respective temperature regimes for ~ 40 h (i.e. 20 h holding period plus 20 h respirometry perdio). Arroyo Seco *O. mykiss* CT_MAX_ increased with increasing temperatures, indicating that these fish are able to adjust their upper thermal limits rapidly. Prologued exposure to these temperature regimes may confer a different response, potentially further increasing CT_MAX_ if the acclimation response was incomplete after 40 h or potentially even decreasing CT_MAX_ if prolonged high temperature exposure had detrimental impacts on fish performance. Even still, this study demonstrates the novel result these fish are capable of rapidly adjusting their thermal tolerance in response to acute temperature changes in this system. By contrast, Piru Creek *O. mykiss* did not adjust their CT_MAX_ based on test temperatures. This indicates that the Arroyo Seco population has greater plasticity in upper critical thermal limits compared to the Piru Creek population. Piru Creek CT_MAX_ matches the maximum CT_MAX_ measured consistently for this species (31 °C)^33,76^, indicating that this population has reached its proverbial “concrete ceiling” for upper thermal tolerance^75^. These results are consistent with the commonly observed tradeoff between upper thermal tolerance and thermal plasticity^53,54^.

### Vulnerability to extirpation from warming

Our results suggest that these two populations will be impacted by rising temperatures in different ways. While Piru Creek *O. mykiss* showed no evidence of limited AAS, FAS, or recovery metrics with increasing temperatures, this population will require sufficient food and oxygen to support their relatively high oxygen requirements for baseline metabolism and exercise recovery. This population will likely be more vulnerable to extirpation from warming if warming coincides with food limitation or low oxygen availability^77–79^. Southern California streams are known to exhibit substantial changes in water levels and invertebrate assemblages during drought years^80^, so Piru Creek and nearby populations may very well be at risk of experiencing these stressors. But, the reduced Q_10_ of RMR across temperatures allows the Piru Creek population to maintain a steady sufficiently high FAS (≥ 3) across a broad range of temperatures (20 °C to 25 °C). On the other hand, the Arroyo Seco population is more likely to face a limitation of FAS with increasing temperatures and likely reach a critical threshold of FAS = 3 around 23 °C (Fig. 6), a temperature that they now experience after a recent fire and debris flow (Fig. 1). Notably, we did not push either population to their functional thermal limits with our experimental temperatures in this study (it was a priority to release all the fish back to the wild and thus test temperatures were intentionally kept below hazardous levels). Further, we do not know whether population differences are due to local adaptation, developmental plasticity and/or acclimatization to local environmental conditions because we did not conduct a controlled, common garden type experiment.

Piru Creek *O. mykiss* have a TSM of 6 °C and maintained a high FAS (> 3) at 25 °C, suggesting they still had sufficient aerobic capacity to thrive at maximum habitat temperatures and are not at immediate risk of extirpation from warm temperatures. The warmest temperature we measured in this stream was 25 °C, and the stream only reached this temperature during the hottest time of day and dropped back down at night. However, it is important to note that this does not necessarily reflect the status of populations in other parts of this watershed (Fig. 8). Piru Creek is located directly below a reservoir and receives constant cold-water inflow year-round. In Santa Paula Creek, a creek in the Lower Santa Clara River watershed within the same subbasin as Piru Creek, *O. mykiss* habitat temperatures have been measured up to 33 °C, exceeding our measured CT_MAX_ temperatures^81^. Sloat and Osterback observed *O. mykiss* feeding up to 29 °C, but they were absent in the same pools when temperatures exceeded 30 °C^81^. In Sespe Creek, another tributary in this watershed, trout have been observed over-summering in isolated pools that reach 28 °C during the hottest time of day, although thermal stratification provides cool refuges from warm surface waters^82^. In another case study in this watershed (Piedra Blanca Creek), *O. mykiss* were observed dead in a drying pool that measured 28 °C^83^, while upstream temperatures reached a maximum of 22 °C from June–October of the same year and never fluctuated by more than 2.5 °C per day. This indicates that although Piru Creek *O. mykiss* do not appear to be in imminent danger of extirpation from warming, the same is not guaranteed for other populations in the same watershed.

Based on stream temperatures during this study (14–18 °C in 2019) Arroyo Seco *O. mykiss* appeared to be buffered against reaching the FAS = 3 threshold of 23 °C. Unfortunately, the Dolan Fire (2020) and subsequent debris flow has since altered the habitat in this stream and temperatures reached 23 °C during the summer of 2022 (Fig. 1). Accordingly, Arroyo Seco *O. mykiss* currently have a TSM of 6.5 °C and a FWT of 0 °C, indicating that they experienced functionally limiting temperatures in 2022. However, vulnerability is a combination of temperature exposure, physiological sensitivity, and adaptive capacity. Here, we tested the physiological sensitivity of these fish under ecologically relevant temperature exposures, but their full acclimation capacity remains uncertain due to our short exposure times. It is possible these fish could improve thermal tolerance and aerobic performance through physiological acclimation processes if they experience these temperatures for longer time periods (i.e., days or weeks). We are also uncertain about the capacity of this population to adapt to these conditions over generations. Both acclimation and adaptation could protect this population from adverse effects of habitat warming.

Stream environments in Southern California are notoriously stochastic and subjected to stressors such as drought and wildfire and *O. mykiss* populations are at risk for potential co-occurring stressors such as hypoxia^82^, food limitation^84^, and predation by non-native species^85^. While populations such as Piru Creek are not in immediate danger of experiencing their thermal limits, they may be at risk of experiencing these other stressors, which can interact with temperature to constrain growth, summer survival, and life history expression^86–88^. Additionally, other southern *O. mykiss* populations risk exceeding upper functional thermal limits in the wild and rely on small pockets of refugia from lethal temperatures and complete habitat drying. Our temperature data from the summer of 2019 indicate that there are cool, stable refugia within all four watersheds that we sampled (Fig. S3). However, manmade barriers and extreme seasonal drying often prevent fish from moving between tributaries of the same watershed, especially in the summer^89^. There is thus a limited ability for fish to behaviorally select their environment. Indeed, genetic analysis of southern *O. mykiss* populations has indicated that tributaries within the same watershed (including Piru Creek, Lion Creek, Sespe Creek, and Santa Paula Creek from the Santa Clara River watershed) are genetically distinct from one another and it is unlikely that they have interbred with one another in the recent past. Our results reinforce the need to integrate population-specific thermal physiology with habitat temperature data in order to understand which populations are most in need of management interventions to improve summer survival (e.g., riparian vegetation restoration to reduce water temperatures, protection from angling, and invasive species removal). Overall, very little is known about the stream temperature heterogeneity, thermal tolerance, or movement for *O. mykiss* at their range limit. It is critical that future studies continue to collect this information for this species as well as other species of conservation concern in order to most effectively protect and restore valuable fish stocks in a changing climate.

## Conclusion

The present study tested the thermal tolerance of two populations of wild *O. mykiss* inhabiting their southern range limit by measuring aerobic performance and upper critical thermal tolerance of fish held and tested at diurnally fluctuating temperatures closely mimicking their natural environment. Our data revealed population-specific functional thermal limitations. The population inhabiting the historically cooler stream (Arroyo Seco) already encounters temperatures that limit aerobic scope. The population inhabiting the warmer stream (Piru Creek) appears to be more resilient to temperature increase, but will need to consume enough food and oxygen to maintain high resting oxygen uptake requirements. Beyond these two populations, several other trout-bearing streams throughout Southern California routinely exceed 25 °C during summer months, indicating that other nearby populations may be more susceptible to warming compared to the Piru Creek population. Taken together, these results reveal population-specific mechanisms of vulnerability to climate change and potential for increased resiliency to thermal stress at the southern range limit. As stream temperatures continue to warm, the survival of fishes inhabiting their range limits will depend on the thermal properties of individual streams and the ability of populations within to physiologically adjust. This highlights the need for population-specific conservation and management strategies, especially broadly distributed fish species that occupy a range of thermal habitats.

## Methods

### O. mykiss populations

Experiments were conducted from August 21–25, 2019 at Piru Creek (34.61691, − 118.74427; Castaic, California, USA) and from September 9–13, 2019 at Arroyo Seco (36.11914, − 121.46904; Greenfield, California, USA; Fig. 1) when temperatures were anticipated to be near peak levels for both populations. Both streams contain robust *O. mykiss* populations and are located near the southern latitudinal limit of this species’ native range. Both the Piru Creek and Arroyo Seco populations have coastal steelhead genetic lineage but have resided for many generations behind man-made barriers to ocean migration^61^. These populations have minimal evidence of genetic introgression with hatchery rainbow trout despite a history of widespread stocking of hatchery rainbow trout in California^61^.

Piru Creek, a tributary to the Santa Clara River, is located further south and is characterized by high and variable temperatures (~ 9–25 °C; Fig. 1 & Fig. S3). The Piru Creek *O. mykiss* population is known as being one of the most southern wild populations that continues to thrive despite warm temperatures, manipulated habitat, introduction of predatory invasive species (e.g., largemouth bass), heavy recreational use, and frequent wildfires. This population is located between two reservoirs (Fig. 1). Downstream, a 61 m dam near the confluence with the mainstem river has blocked this population from ocean migration since 1955. Upstream, a 118 m dam separates this population from the headwaters. This reach is perennially wetted due to continuous outflow from the upstream reservoir.

Arroyo Seco, a tributary to the Salinas River, is located ~ 300 km north of Piru Creek. We sampled fish from a spring-fed, perennially wetted reach near the headwaters. This population typically experiences cooler temperatures (~ 5–23 °C; Fig. 1) than Piru Creek and is located in a more pristine, unmanipulated habitat. Still, this population has been earmarked for conservation due to inhabiting “stochastic” conditions relative to the rest of the range^89,90^. This population coexists with invasive species (e.g., Sacramento pikeminnow and brown trout) and this area is at high risk for wildfire. In fact, the Dolan Fire (Aug. 18–Dec. 31, 2020) and subsequent debris flow in 2021 prevented us from retrieving our temperature loggers, so we do not have year-round temperature data for this site (Fig. 1). There are no year-round complete barriers to anadromy for this population, but there are six barriers that partially or temporally block upstream migration to our sample site (Fig. 1). On the upstream end is a natural chute where high flows are required for fish to ascend. Further downstream there are three road crossings, a diversion dam, and a section of the stream that has sustained damage from gravel mining. Fish passage from the ocean is only possible during high flow events. These barriers, combined with typical low flows due to groundwater extraction in the lower watershed, make steelhead migration extremely unlikely for this population.

### Field methodology

All experiments were conducted streamside no more than 3.2 km from fish collection sites. A temporary tank system was constructed at each site. Water was pumped directly from the creek into 2 header tanks (531 L and 102 L) equipped with Smart One Easy Plug Axial Heaters (Pentair Aquatic Eco-Systems, Inc., Minneapolis, USA) to heat water to test temperatures. Water was pumped from the header tanks into an acclimation tank (102 L) and respirometry tanks (six tanks, 102 L each). Power was supplied from two portable inverter generators (EU7000IS and EU3000IS; Honda Motor Company Ltd, Japan).

All experimental procedures were approved by the University of California Santa Barbara Institutional Animal Care and Use Committee and experiments were performed in accordance with the relevant guidelines and regulations. All experiments were non-lethal and all fish were returned to the stream upon completion.

### Temperature treatments

Juvenile *O*. *mykiss* (Piru: n = 32, body mass = 23.8 ± 2.45 g; Arroyo Seco 2019: n = 34, body mass = 27.4 ± 4.08 g) were collected via electrofishing using settings specific to the water conductivity at each location. Typically, fish thermal tolerance studies are conducted by assessing performance of fish either acutely exposed or acclimated to two or more static temperatures. We instead allowed fish to experience natural diurnal fluctuations that they typically experience in their environment. Temperature logger data revealed diurnal temperature fluctuations between 3 and 5 °C at both of our study sites during experiments (Piru: 17.5–22.5 °C; Arroyo Seco: 14.5–18 °C), making it less ecologically relevant to test fish held at static temperatures. Instead, we allowed our holding and respirometry tank temperatures to fluctuate along with natural diurnal temperature cycles in order to best simulate each population’s natural thermal habitat and to mimic what an increase in stream temperature would most likely look like for these fish. Fish were held at one of three temperature treatments: 1: ambient stream temperature, 2: 2–3 °C above ambient stream temperature (+ 3 °C), and 3: 5 °C above ambient stream temperature (+ 5 °C) for 19–23 h before respirometry (see Table 1). This fairly brief overnight temperature exposure duration was selected to represent a short-term, ecologically relevant thermal stress for the fish.

Fish were always collected the day before their experiment began. After collection, fish were held overnight at their treatment temperature before respirometry experiments began (mean duration: 20 h). For temperature treatments above ambient, fish were placed in the acclimation tank at ambient stream temperature and ramped up to their treatment temperature at a rate of 2 °C per hour. Dissolved oxygen in the acclimation tank was maintained at above 90% saturation at all times. During the day, temperature and dissolved oxygen were monitored using an OxyGuard Handy Polaris Dissolved Oxygen Meter (OxyGuard, Denmark). Temperatures were continuously recorded in the stream and acclimation tanks using HOBO Waterproof Bluetooth Pendant Temperature Data Loggers (Onset Computer Corporation, Bourne, USA). Fish were not fed during acclimation to ensure that they were in a post-absorptive state during experiments and the holding and respirometry tanks were covered with fine mesh shade cloth and situated underneath shade canopies so that food items could not enter the tanks from above.

### Respirometry

Once fish completed the overnight ~ 20 h temperature treatment, oxygen uptake rate (MO_2_) measurements were taken using intermittent flow respirometry beginning the following morning. We used twelve respirometry chambers (volumes 1.4 L, 1.8 L, and 2.1 L) constructed from airtight plastic containers (Lock & Lock, Seoul, South Korea) and tested 1 fish in each chamber. Within each chamber, we aimed for a water volume: fish mass ratio of 20:1 but fish were smaller than anticipated and the mean ratio was 87:1 (assuming 1 kg fish = 1 L water). Two respirometers were placed in each of the six respirometry tanks. Each chamber was fitted with a FireStingO2 robust oxygen probe (PyroScience, Germany) on a recirculation loop that moved water through the chamber at all times using a CompactON 300-L h^−1^ pump (Eheim, Germany). Each chamber was also connected to a Universal 300-L h^−1^ pump (Eheim, Germany) that intermittently flushed oxygenated water through the chamber from the surrounding tank to ensure that the fish never experienced dissolved oxygen levels of less than 70% air saturation. At Piru, MO_2_ was measured over 4-min measurement cycles, separated by 6-min flush cycles. At Arroyo Seco, MO_2_ was measured over 5–12-min measurement cycles, followed by 10-min flush cycles.

Maximum metabolic rate (MMR) was measured first, followed by exercise recovery, followed by resting metabolic rate (RMR) and standard metabolic rate (SMR). To induce (MMR), fish were chased individually for 3 min in a bucket using quick hand movements and gentle caudal fin contact to induce burst swimming. Fish were then immediately netted and exposed to air for 1 min^91^. This method has been found to elicit the same MMR results as other chase methods (e.g. a chase to exhaustion) while having greater statistical power^91^. Fish were then placed in respirometers and MO_2_ measurements were taken continuously over 20 h. Shade cloth was placed over the respirometer tanks to minimize disturbance and direct sun exposure during MO_2_ measurements. For five fish at Piru, MMR measurements were compromised and MMR was re-measured the following day.

After 20 h of respirometry, fish were removed from the chambers, weighed, and measured for fork length and standard length. Bacterial respiration was measured for 1 h by measuring MO_2_ in each chamber after the fish were removed and was determined to be negligible.

### Critical thermal maximum

Upper thermal tolerance was assessed using CT_MAX_ tests conducted post-respirometry. At this point, the fish had been exposed to their treatment temperatures for ~ 40 h. To account for possible effects of diurnal light cycle on CT_MAX_^31^, all fish were tested at the same time of day. Fish were transferred to an aerated cooler containing water at their corresponding holding temperature for the time of day of the experiment (see Table 1). Dissolved oxygen in the cooler remained > 90% throughout CT_MAX_ testing. Water temperature was increased by 0.3 °C per minute^60^ by circulating heated water through a stainless-steel coil and dipping the coil in and out of the water. Temperature at the moment each fish lost equilibrium was recorded, at which point the individual fish were immediately transferred to a bucket and recovered back to ambient stream temperature. After CT_MAX_ testing and recovery, fish were released back to their collection site.

### Data analysis and statistics

All data analysis was conducted using RStudio version 1.4.423. All statistical tests used a significance level of α = 0.05. First, decreases in dissolved oxygen from each MO_2_ measurement cycle were plotted and inspected visually for linearity. For MMR measurements, regressions with an R^2^ of ≥ 0.8 were included in our analysis (Piru: n = 25, Arroyo Seco 2019: n = 23). For all other measurements, regressions with an R^2^ of less than 0.75 were excluded from further analysis. Fish with ≥ 75% of regressions with an R^2^ above 0.75 were included in RMR analysis (Piru: n = 19, Arroyo Seco: n = 18). MO_2_ (mgO_2_ kg^−1^ min^−1^) for each measurement cycle was calculated using the slope of each regression using the following equation: MO_2_ = (slope × (v_R_ – m))/m × (m/0.03)^(1−scaling exponent)^, where v_R_ is the respirometer volume and m is the fish body weight in kg. All MO_2_ measurements were scaled to a common body size of 30 g using data-generated scaling exponents of 0.79 for MMR and 0.67 for all other MO_2_ measurements. These exponents were generated by fitting linear regressions to the log–log relationship between body size and MO_2_ (Fig. S2).

MMR was calculated using the steepest 120 s slope from the first measurement cycle (taken immediately after the chase)^91^. All MMR measurements occurred during the first measurement cycle post-chase. Both SMR and RMR are used to quantify the oxygen uptake of a fish at rest. We define SMR as the minimum oxygen uptake rate across the 20-h respirometry period^92^. Standard metabolic rate (SMR) was calculated by averaging the lowest ten MO_2_ measurements (after excluding the five absolute lowest^92^). RMR includes all MO_2_ measurements taken while the fish is at rest post-exercise recovery (Fig. 3). All measurements taken after MO_2_ reached 20% of SMR (after EPOC was complete, typically ~ 5–8 h after MMR) were considered “post-recovery” and included in the RMR calculations (Fig. S1). By defining RMR this way, we were able to measure the oxygen uptake rate of at-rest fish at each temperature the fish experienced during their respective diurnal temperature cycles to account for possible effects of this natural acute temperature change on resting metabolism.

Due to the diurnal temperature fluctuations, we obtained RMR measurements for fish at eight different temperatures at Piru and six different temperatures at Arroyo Seco (with temperatures rounded to the nearest 1 °C). To be included in the RMR estimates for a given temperature, individual fish were required to have at least 3 RMR measurements taken at that temperature. In order for a given temperature to be included in our RMR analysis, at least three fish had to have RMR estimates at that temperature. The effect of temperature on RMR and on *ln*(RMR) was modeled for each population using the R package lme4^93^ to fit linear mixed effects models with temperature treatment group included as an additional fixed effect and individual fish ID included as a random effect. The best fit models were selected using BIC. Type III ANOVAs (R package: “car”)^94^ revealed that the interaction between the fixed effects was insignificant for both populations, so the interaction term was dropped and type II ANOVAs were used to test these relationships. The effect of temperature treatment was also found to be insignificant, so this effect was dropped from the final models. Student’s t-tests were used to compare RMR measurements at each of the four common temperatures between populations.

We calculated aerobic scopes for each fish using RMR measurements taken at temperatures as close as possible to MMR temperatures. While we are aware that using RMR instead of standard metabolic rate (SMR) could underestimate aerobic scope, fish in the present study recovered quickly and were very calm in the chambers with minimal spontaneous activity. RMR values were thus very close to SMR values, and underestimates of aerobic scope are likely negligeable. Absolute aerobic scope (AAS) was calculated by subtracting RMR from MMR. Factorial aerobic scope (FAS) was calculated by dividing MMR by RMR. MMR, AAS, and FAS results were analyzed using one-way ANOVAs to compare across temperature treatments for each population. CT_MAX_ results were analyzed using one-way ANOVAs and post-hoc Tukey’s tests to compare between temperature treatments for each population and using Mann–Whitney *U* tests to compare between populations for two common temperature treatments (Piru Ambient & Arroyo Seco + 3 °C; Piru + 3 °C & Arroyo Seco + 5 °C).

MO_2_ measurements taken before fish reached 20% of SMR were considered part of each fish’s recovery period (Fig. S1). We first assessed exercise recovery by quantifying the duration and magnitude of EPOC. In other words, we measured the time it took for each fish to reach 20% of SMR after MMR was measured and the amount of oxygen consumed during this time using methods described in Zhang et al.^95^. EPOC magnitude and duration were compared between temperature treatments for each population using one-way ANOVAs and between populations using Student’s t-tests. We then assessed MO_2_ as a percent of MMR (%MMR) over the first hour of exercise recovery at 10, 20, 30, 40, 50, and 60 min after the chase. Changes in %MMR over time were assessed using a linear mixed effect model with the variables time, population, and temperature treatment as fixed effects and fish ID as a random effect. A type III ANOVA revealed that there were no significant interactions between fixed effects, so the interaction terms were dropped and a type II ANOVA was used to analyze this relationship. There was also no significant effect of temperature treatment on changes in %MMR over time, so this effect was dropped from the final model. Post-hoc analysis was conducted using least-squares means (R package: “emmeans”)^96^.

### Habitat temperature data

Stream temperature was recorded using HOBO Waterproof Bluetooth Pendant Temperature Data Loggers (Onset Computer Corporation, Bourne, USA), HOBO Dissolved Oxygen Loggers (Onset Computer Corporation, Bourne, USA), and miniDOT Loggers (Precision Measurement Engineering, Vista, USA). All loggers recorded water temperature measurements continuously every 10 min (miniDOT Loggers) or 15 min (HOBO Pendants and Dissolved Oxygen Loggers) minutes for the duration of their deployment. Temperature data were obtained from Piru Creek from June 2019–2022. Initial loggers placed at Arroyo Seco during the time of this study were destroyed in a fire and subsequent debris flow. Therefore, temperature data was obtained from Arroyo Seco from May–November 2022. To compare Piru Creek to other streams within the Santa Clara River watershed, we collected temperature data from Lion Creek (34.54338°N, − 119.16372°W) and Piedra Blanca Creek (34.58515°N, − 119.16543°W) in 2021–2022 and we obtained open access daily temperature summary data from the NorWesT Reigonal Database^97^ for Santa Paula Creek (34.42763°N, − 119.09089°W) from 2008 to 2011 and Sespe Creek (34.44492°N, − 118.92715°W) from 2008 to 2013. Additionally, we collected temperature data from 10 additional trout-bearing streams throughout the Los Padres National Forest between June and October of 2019 to compare thermal regimes with Piru Creek and Arroyo Seco. Temperature maxima and minima were considered the maximum and minimum daily stream temperatures that occurred during 3 or more days per sample year during the summer months (June–September). All temperature measurements were rounded to the nearest degree for this analysis.

### Supplementary Information

Supplementary Information.

## Data Availability

All data collected and analyzed in the present study are available from the corresponding author upon reasonable request.
