# Supplementary material for: Thermal tolerance and vulnerability to warming differ between populations of wild Oncorhynchus mykiss near the species’ southern range limit
Source: Sci Rep. 2023 Sep 4;13:14538. doi: 10.1038/s41598-023-41173-7 (PMC10477306; doi:10.1038/s41598-023-41173-7)

**Supplementary Information**

**Thermal tolerance and vulnerability to warming differ between populations of wild *Oncorhynchus mykiss* near the species’ southern range limit**

Dressler, T.L.^1^, Han Lee, V.^1^, Klose, K.^2^, Eliason, E.J.^1*^

^*^Corresponding Author: eliason@ucsb.edu

^1^Department of Ecology, Evolution, and Marine Biology, University of California, Santa Barbara, CA, 93106, USA

^2^U.S. Forest Service, Los Padres National Forest, 1980 Old Mission Drive, Solvang, CA 93463, USA

**Table S1.** Critical Thermal Maximum (CT_MAX_) for each population and temperature treatment. All values are presented as mean ± SEM. Differing letters indicate statistically significant differences within populations (one-way Anova; p<0.05; Piru Creek: a,b; Arroyo Seco: x,y).

| ***Population*** | ***Temperature Treatment*** | ***Temperature Range (°C)*** | ***CT_MAX_ Start Temperature (°C)*** | ***Mean ± SEM***  ***CT_MAX_*** | ***Body mass (g)*** | ***n*** | ***df*** | ***F-value*** | ***p-value*** |
| --- | --- | --- | --- | --- | --- | --- | --- | --- | --- |
| Piru Creek | Ambient | 17-22 | 20 | 30.29 ± 0.62^a^ | 30.5 ± 2.36 | 11 | 2 | 2.680 | 0.086 |
|  | +3°C | 20-24 | 24 | 31.30 ± 0.02^a^ | 23.5 ± 5.87 | 11 |  |  |  |
|  | +5°C | 22-26 | 25 | 31.43 ± 0.06^a^ | 15.3 ± 1.86 | 9 |  |  |  |
| Arroyo Seco | Ambient | 14-19 | 16 | 27.49 ± 0.78^x^ | 29.0 ± 3.05 | 11 | 2 | 4.711 | **0.017** |
|  | +3°C | 17-21 | 19 | 29.83 ±0.24^x^ | 34.3 ± 13.02 | 10 |  |  |  |
|  | +5°C | 19-24 | 22 | 29.52 ± 0.57^y^ | 24.3 ± 4.27 | 10 |  |  |  |

**Table S2.** Critical Thermal Maximum (CT_MAX_) for each population at each common temperature treatment. All values are presented as mean ± SEM. Differing letters indicate statistically significant differences between populations (Mann-Whitney U; p<0.05).

| ***Temperature Treatment*** | ***Population*** | ***CT_MAX_ Start Temperature (°C)*** | ***Mean ± SEM***  ***CT_MAX_*** | ***Body mass (g)*** | ***n*** | ***W*** | ***p-value*** |
| --- | --- | --- | --- | --- | --- | --- | --- |
| 17-22°C | Piru Creek | 20 | 30.29 ± 0.62^a^ | 30.5 ± 2.36 | 11 | 92 | **0.007** |
|  | Arroyo Seco | 19 | 29.83 ±0.24^b^ | 34.3 ± 13.02 | 10 |  |  |
| 20-24°C | Piru Creek | 24 | 31.30 ± 0.02^x^ | 23.5 ± 5.87 | 11 | 4.711 | **0.013** |
|  | Arroyo Seco | 22 | 29.52 ± 0.57^y^ | 24.3 ± 4.27 | 10 |  |  |

**Table S3.** Statistical outputs from each of the linear mixed models fitted to the relationship between resting metabolic rate (RMR) and temperature. Temperature refers to the test temperature at the time of each MO_2_ measurement. Treatment Group refers to holding temperature regimes (Ambient, +3°C, +5°C). Best fit models are highlighted in grey.

| ***Population*** | ***Dependent Variable*** | ***Fixed Effect*** | ***X^2^*** | ***Df*** | ***p-value*** | ***AIC*** | ***BIC*** |
| --- | --- | --- | --- | --- | --- | --- | --- |
| Piru Creek | RMR | Temperature | 3.955 | 1 | **0.047** | 188.37 | 205.76 |
|  |  | Treatment Group | 1.121 | 2 | 0.571 |  |  |
|  |  | Temperature: Treatment Group | 1.225 | 2 | 0.542 |  |  |
|  | RMR | Temperature | 6.346 | 1 | **0.012** | 185.58 | 198.62 |
|  |  | Treatment Group | 1.829 | 2 | 0.401 |  |  |
|  | RMR | Temperature | 14.855 | 1 | **<0.001** | 183.38 | 192.08 |
|  | *ln*(RMR) | Temperature | 4.676 | 1 | **0.031** | 0.56 | 17.95 |
|  |  | Treatment Group | 0.645 | 2 | 0.724 |  |  |
|  |  | Temperature: Treatment Group | 0.560 | 2 | 0.756 |  |  |
|  | *ln*(RMR) | Temperature | 6.578 | 1 | **0.010** | -2.88 | 10.16 |
|  |  | Treatment Group | 3.517 | 2 | 0.172 |  |  |
|  | *ln*(RMR) | Temperature | 15.045 | 1 | **<0.001** | -3.50 | 5.20 |
| Arroyo Seco | RMR | Temperature | 3.725 | 1 | 0.054 | 61.63 | 77.24 |
|  |  | Treatment Group | 2.235 | 2 | 0.327 |  |  |
|  |  | Temperature: Treatment Group | 2.633 | 2 | 0.268 |  |  |
|  | RMR | Temperature | 10.630 | 1 | **0.001** | 60.05 | 71.76 |
|  |  | Treatment Group | 3.567 | 2 | 0.168 |  |  |
|  | RMR | Temperature | 33.491 | 1 | **<0.001** | 59.44 | 67.25 |
|  | *ln*(RMR) | Temperature | 9.228 | 1 | **0.002** | 18.35 | 33.96 |
|  |  | Treatment Group | 4.890 | 2 | 0.087 |  |  |
|  |  | Temperature: Treatment Group | 4.272 | 2 | 0.118 |  |  |
|  | *ln*(RMR) | Temperature | 9.928 | 1 | **0.002** | 18.20 | 29.91 |
|  |  | Treatment Group | 4.807 | 2 | 0.090 |  |  |
|  | *ln*(RMR) | Temperature | 30.345 | 1 | **<0.001** | 18.71 | 26.52 |

**Table S4.** Resting Metabolic Rate (RMR) of *O. mykiss* from each of the Piru Creek and Arroyo Seco populations measured at 4 common temperatures. Represented are mean and standard error values and t-test results comparing populations at each temperature.

| ***Temperature (°C)*** | ***Population*** | ***Mean ± SEM***  ***RMR*** | ***n*** | ***df*** | ***t-value*** | ***p-value*** |
| --- | --- | --- | --- | --- | --- | --- |
| 18 | Piru Creek | 2.95 ± 0.31 | 6 | 9 | -4.54 | **0.001** |
|  | Arroyo Seco | 1.29 ± 0.14 | 5 |  |  |  |
| 19 | Piru Creek | 2.69 ± 0.15 | 6 | 16 | -3.92 | **0.002** |
|  | Arroyo Seco | 1.70 ± 0.18 | 12 |  |  |  |
| 20 | Piru Creek | 3.58 ± 0.43 | 11 | 11.77 | -4.00 | **0.002** |
|  | Arroyo Seco | 1.77 ± 0.13 | 10 |  |  |  |
| 21 | Piru Creek | 3.64 ± 0.44 | 11 | 12.56 | -3.65 | **0.003** |
|  | Arroyo Seco | 1.95 ± 0.16 | 8 |  |  |  |

**Table S5.** Statistical outputs from linear mixed models fitted to the relationship between percent maximum metabolic rate (MMR) and time post-MMR. The best fit model is highlighted in grey.

| ***Dependent Variable*** | ***Fixed Effect*** | ***X^2^*** | ***Df*** | ***p-value*** | ***BIC*** |
| --- | --- | --- | --- | --- | --- |
| Percent MMR | Time post-MMR | 0.781 | 5 | 0.974 | 34.67 |
|  | Population | 10.950 | 1 | **<0.001** |  |
|  | Temp Treatment | 3.04 | 2 | 0.165 |  |
|  | Time: Population | 3.185 | 5 | 0.674 |  |
|  | Time: Temp Treatment | 12.345 | 10 | 0.246 |  |
|  | Population: Temp Treatment | 0.815 | 1 | 0.400 |  |
|  | Time: Population: Temp Treatment | 3.624 | 4 | 0.433 |  |
| Percent MMR | Time post-MMR | 37.712 | 5 | **<0.001** | -119.42 |
|  | Population | 49.393 | 1 | **<0.001** |  |
|  | Temp Treatment | 3.900 | 2 | 0.083 |  |
| Percent MMR | Time post-MMR | 37.812 | 5 | **<0.001** | -133.76 |
|  | Population | 45.626 | 1 | **<0.001** |  |

**Table S6.** Summary of all temperature data collected throughout the Los Padres National Forest. Temperatures shown represent the maximum, minimum, and average temperatures for each location during the summer months (June-September) during the years each logger was deployed.

| **Stream Name** | **Watershed** | **Location** | **Maximum Summer Temperature** | **Minimum Summer Temperature** | **Mean Summer Temperature** | **Summer Diurnal Fluctuation Range** | **Years Measured** |
| --- | --- | --- | --- | --- | --- | --- | --- |
| Arroyo Seco | Salinas River | 36.11914,  -121.46904 | 23°C | 13°C | 19°C | 1-5°C | 2022 |
| Lion Creek | Santa Clara River | 34.54338,  -119.16372 | 20°C | 13°C | 15°C | 1-6°C | 2019, 2021, 2022 |
| Piedra Blanca Creek | Santa Clara River | 34.58515,  -119.16543 | 22°C | 13°C | 18°C | 0-2°C | 2021 |
| Piru Creek | Santa Clara River | 34.62655,  -118.74397 | 25°C | 15°C | 20°C | 1-4°C | 2019, 2020, 2021 |
| Sespe Creek | Santa Clara River | 34.44492,  -118.92715 | 31°C | 17°C | 24°C | 2-11°C | 2008, 2009, 2013 |
| Santa Paula Creek | Santa Clara River | 34.42763,  -119.09089 | 28°C | 15°C | 21°C | 1-10°C | 2008, 2009, 2010 |
| Davey Brown Creek | Santa Maria River | 34.73486,  -119.96602 | 18°C | 12°C | 15°C | 0-2°C | 2019 |
| Manzana Creek | Santa Maria River | 34.76897,  -119.93607 | 27°C | 13°C | 20°C | 6-11°C | 2019 |
| Munch Creek | Santa Maria River | 34.74594,  -119.92533 | 21°C | 12°C | 17°C | 3-6°C | 2019 |
| Alder Creek | Santa Ynez River | 34.48329,  -119.49618 | 22°C | 14°C | 17°C | 2-6°C | 2019 |
| Bear Creek | Santa Ynez River | 34.5257  -119.86739 | 18°C | 14°C | 16°C | 0-4°C | 2019 |
| Mono Creek | Santa Ynez River | 34.54117  -119.62472 | 20°C | 15°C | 18°C | 0-6°C | 2019 |
| Matilija Creek | Ventura River | 34.52345,  -119.40136 | 28°C | 16°C | 21°C | 3-10°C | 2019 |
| Murrieta Creek | Ventura River | 34.50331,  -119.38805 | 23°C | 17°C | 19°C | 3-5°C | 2019 |
| North Fork Matilija Creek | Ventura River | 34.50606,  -119.27772 | 25°C | 17°C | 20°C | 4-8°C | 2019 |
| Upper North Fork Matilija Creek | Ventura River | 34.51057,  -119.38292 | 29°C | 14°C | 21°C | 2-14°C | 2019 |

**Table S7.** Respirometry methods checklist (adapted from Killen et al. 2021).

|  |  |
| --- | --- |
| Criterion and Category | **Response** |
|  |  |
| EQUIPMENT, MATERIALS, AND SETUP |  |
| Body mass of animals at time of respirometry | 5-118 g |
| Volume of empty respirometers | 1.4 L, 1.8 L, 2.1 L |
| How chamber mixing was achieved | Recirculation pump (Eheim CompactON 300-L h^-1^) |
| Ratio of net respirometer volume (plus any associated tubing in mixing circuit) to animal body mass | 87:1 (mean) |
| Material of tubing used in any mixing circuit | PVC |
| Confirm volume of tubing in any mixing circuit was included in calculations of oxygen uptake | confirmed |
| Material of respirometer (e.g. glass, acrylic, etc.) | plastic |
| Type of oxygen probe and data recording | FirestingO2 |
| Sampling frequency of water dissolved oxygen | Every 1-2 seconds |
| Describe placement of oxygen probe (in mixing circuit or directly in chamber) | In mixing circuit |
| Confirm that chamber returned to normoxia during flushing | Confirmed |
| Timing of flush/closed cycles | Piru: 4min closed, 6min flush  Arroyo Seco: 5-12min closes, 10min flush |
| Wait (delay) time excluded from closed measurement cycles | Piru: 0 s  Arroyo Seco: 30 s |
| Frequency and method of probe calibration (for both 0 and 100% calibrations) | Once at the beginning of each field excursion. |
|  |  |
| MEASUREMENT CONDITIONS |  |
| Temperature during respirometry | Ranged from 14-25°C between treatments, fluctuated by 4-5°C within treatments. |
| How temperature was controlled | Pentair Smart One Easy Plug Axial Heaters |
| Photoperiod during respirometry | Natural (experiments conducted outside) |
| If (and how) ambient water bath was cleaned and aerated during measurement of oxygen uptake | Continuous flow of water in from the stream into header tank. Header tank equipped with electric aerators. |
| Total volume of header tank. | 581 L |
| Minimum water oxygen dissolved oxygen reached during closed phases | 70% |
| State whether chambers were visually shielded from external disturbance | Yes, shade cloth placed over respirometry tanks |
| Duration of animal fasting before placement in respirometer | ~20 hours |
| Duration of all trials combined (number of days to measure all animals in the study) | 6 days of respirometry |
| Acclimation time to the laboratory (or time since capture for field studies) before respirometry measurements | 19-23 hours in holding tank |
|  |  |
| BACKGROUND RESPIRATION |  |
| State whether background microbial respiration was measured and accounted for, and if so, method used | Background measured in empty chambers after each round of respirometry for 1 hour. |
| State how changes in background respiration were modelled over time (e.g. linear, exponential, parallel measures) | Background was negligible, therefore not modelled |
| Level of background respiration (e.g. as a percentage of SMR) | 0% SMR |
|  |  |
| STANDARD OR ROUTINE METABOLIC RATE |  |
| Time period, within a trial, over which oxygen uptake was measured (e.g. number of hours) | 20 hours total (including MMR, recovery, SMR/RMR) |
| Value taken as SMR/RMR (e.g. quantile, mean of lowest 10 percent, mean of all values) | SMR: lowest 10 measurements  RMR: mean of all values at a given temperature |
| Whether any time periods were removed from calculations of SMR/RMR | MMR was measured first and recovery period was removed from SMR/RMR calculations. |
| r^2^ threshold for slopes used for SMR/RMR | 0.75 |
|  |  |
| MAXIMUM METABOLIC RATE |  |
| When MMR was measured in relation to SMR (i.e. before or after) | before |
| Method used | 3 min chase, 1 min air exposure |
| Value taken as MMR (e.g. the highest rate of oxygen uptake value after transfer, average of highest values) | Steepest 120 s slope after transfer |
| If MMR measured post-exhaustion, time until transfer to chamber after exhaustion or time to start of oxygen uptake recording | < 30 s |
| Duration of slopes used to calculate MMR | Steepest 120 s slope from a 4-minute measurement slope |
| How absolute aerobic scope and/or factorial aerobic scope is calculated (i.e. using raw SMR and MMR, allometrically mass-adjusted SMR and MMR, or allometrically mass-adjusting aerobic scope itself) | Allometrically mass-adjusted RMR and MMR. |
|  |  |
| DATA HANDLING AND STATISTICS |  |
| Sample size |  |
| How oxygen uptake rates were calculated (software or script, equation, units, etc.) | Respirometry Performances R package |
| Confirm that volume (mass) of animal was subtracted from respirometer volume when calculating oxygen uptake rates | confirmed |
| State whether analyses accounted for variation in body mass and describe any allometric mass-corrections or adjustments | Data-generated scaling exponents were used for MMR and RMR/SMR and all MO_2_ measurements were scaled to a common body size of 30 g. |

**Figure S1.** A representative MO_2_ trace of a single fish over the duration of a respirometry trial. Circular points represent MO_2_ measurements. The large yellow point at time 0 represents maximum metabolic rate (MMR). The green dotted line represents the duration to reach 50% of MMR (Time_MMR50_). The red horizontal line represents standard metabolic rate (SMR) and the red points represent the values that were averaged to calculate SMR. The bold dashed line represents the time that MO2 reached 20% of SMR. All points before the 20% SMR line were considered part of the fish’s recovery period. The area under the curve between MMR and 20% SMR, shown in gray, was considered excess post-exercise oxygen consumption (EPOC). All points after the 20% SMR line were considered measurements of resting metabolic rate (RMR).


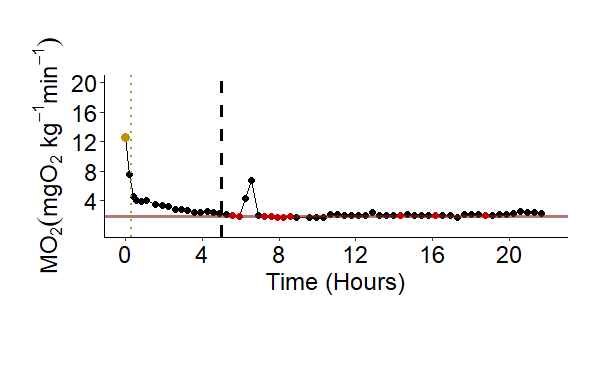


**MMR**

**20%**

**SMR**

**RMR**

**EPOC**

**SMR**

**Time_MMR50_**

**Figure S2.** Log-log plots of the relationship between body mass and MMR (panel A) and RMR (panel B) with Piru Creek trout shown in orange and Arroyo Seco trout shown in blue.


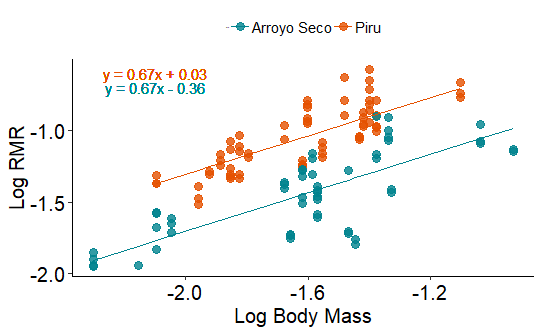

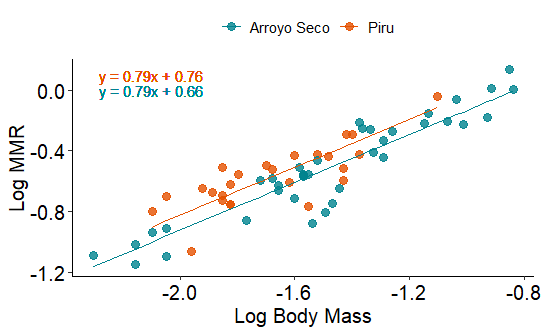


**Figure S3:** Temperature data (collected every 15 minutes) and stream locations for HOBO Dissolved Oxygen and HOBO Pendant loggers placed in 4 separate watersheds during the summer of 2019 (Santa Maria River: blue, Santa Clara River: green, Santa Ynez River: red, Ventura River, purple).


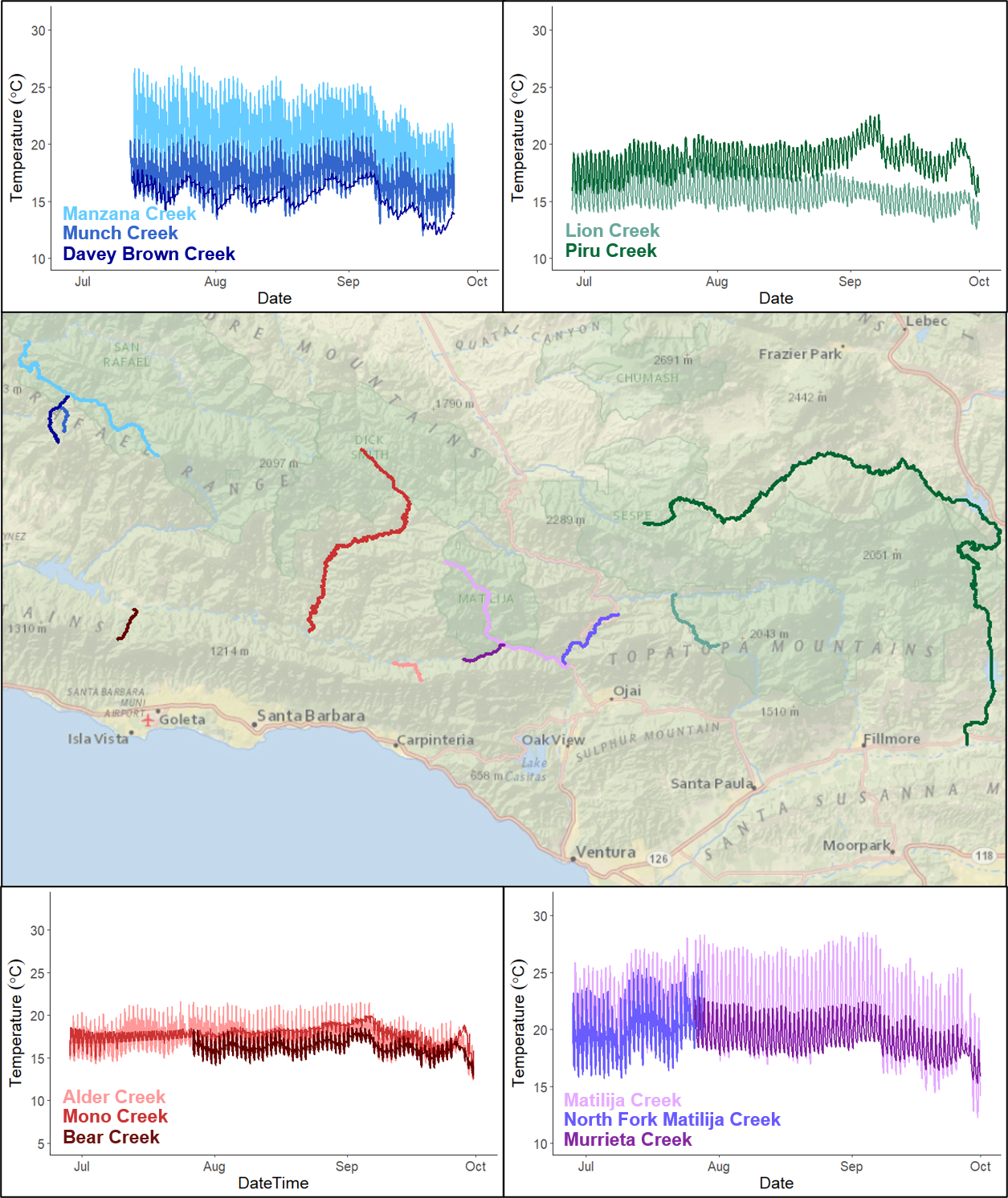

Supplement: Supplementary file 1 — Supplementary Information. [file 41598_2023_41173_MOESM1_ESM.docx]
